# Inhibition of CDK9 attenuates atherosclerosis by inhibiting inflammation and phenotypic switching of vascular smooth muscle cells

**DOI:** 10.18632/aging.202998

**Published:** 2021-06-08

**Authors:** Shushi Huang, Wu Luo, Gaojun Wu, Qirui Shen, Zaishou Zhuang, Daona Yang, Jinfu Qian, Xiang Hu, Yan Cai, Nipon Chattipakorn, Weijian Huang, Guang Liang

**Affiliations:** 1 Chemical Biology Research Center, School of Pharmaceutical Sciences, Wenzhou Medical University, Wenzhou, Zhejiang 325035, China; 2 Department of Cardiology, The First Affiliated Hospital, Wenzhou Medical University, Wenzhou, Zhejiang 325035, China; 3 Affiliated Cangnan Hospital, Wenzhou Medical University, Cangnan, Zhejiang 325000, China; 4 Department of Endocrinology, The First Affiliated Hospital, Wenzhou Medical University, Wenzhou, Zhejiang 325035, China; 5 Cardiac Electrophysiology Research and Training Center, Faculty of Medicine, Chiang Mai University, Chiang Mai 50200, Thailand; 6 School of Pharmaceutical Sciences, Hangzhou Medical College, Hangzhou, Zhejiang 311399, China

**Keywords:** atherosclerosis, CDK9, pharmacological inhibition, inflammation, phenotypic switching, vascular smooth muscle cells

## Abstract

Background: Recent studies have demonstrated a key role of vascular smooth muscle cell (VSMC) dysfunction in atherosclerosis. Cyclin-dependent kinases 9 (CDK9), a potential biomarker of atherosclerosis, was significantly increased in coronary artery disease patient serum and played an important role in inflammatory diseases. This study was to explore the pharmacological role of CDK9 inhibition in attenuating atherosclerosis.

Methods: A small-molecule CDK9 inhibitor, LDC000067, was utilized to treat the high fat diet (HFD)-fed ApoE^-/-^ mice and human VSMCs.

Results: The results showed that inflammation and phenotypic switching of VSMCs were observed in HFD-induced atherosclerosis in ApoE^-/-^ mice, which were accompanied with increased CDK9 in the serum and atherosclerotic lesions where it colocalized with VSMCs. LDC000067 treatment significantly suppressed HFD-induced inflammation, proliferation and phenotypic switching of VSMCs, resulting in reduced atherosclerosis in the ApoE^-/-^ mice, while had no effect on plasma lipids. Further *in vitro* studies confirmed that LDC000067 and siRNA-mediated CDK9 knockdown reversed ox-LDL-induced inflammation and phenotypic switching of VSMCs from a contractile phenotype to a synthetic phenotype via inhibiting NF-κB signaling pathway in human VSMCs.

Conclusion: These results indicate that inhibition of CDK9 may be a novel therapeutic target for the prevention of atherosclerosis.

## INTRODUCTION

Coronary artery disease (CAD) is the main cause of mortality and morbidity worldwide, and atherosclerosis is the most important single causal factor of CAD [1, 2]. Atherosclerosis is a multifactorial disease involving multicellular dysfunctional responses, such as endothelial cell injury, macrophage infiltration, foam cell formation, and vascular smooth muscle cell (VSMC) proliferation and migration [3, 4]. Particularly, VSMCs had demonstrated a prominent role in atherosclerosis [5, 6]. Previously, many studies focused on the apoptosis of VSMCs in atherosclerosis. Interestingly, more recent studies have identified the existence of functional and phenotypic plasticity of VSMCs in atherosclerosis progress [7, 8]. For example, after uptake of oxidized-low density lipoprotein (ox-LDL) by VSMCs, the expression of inflammatory factors was up-regulated accompanied with switching of VSMCs from a contractile phenotype to an adverse proliferative phenotype, which play a dominant role in atherosclerosis progress [9]. However, the underlying mechanisms involved in ox-LDL-induced inflammatory responses and phenotypic switching in VSMCs remain to be elucidated.

Cyclin-dependent kinases (CDKs), a group of serine/threonine protein kinases that are activated by cyclin, are important factors in cell cycle regulation [10]. Among CDKs, CDK9 has been reported to be involved in many chronic disease pathological processes. Abnormal upregulation of CDK9 expression has been demonstrated in the progress of diseases such as acquired immunodeficiency syndrome [11], osteoarthritis [12] and cardiac hypertrophy [13]. More interestingly, CDK9 was indispensable in many inflammatory diseases, where an increased interaction between CDK9 and nuclear factor-κB (NF-κB) and subsequent activation of downstream signals were observed [14]. It has been reported that CDK9 was highly increased in the serum of patients with atherosclerosis [15]. However, the role of CDK9 in atherosclerosis is incompletely illuminated, and it remains unclear if pharmacological inhibition of CDK9 can be developed as a therapeutic strategy for atherosclerosis.

In this study, atherosclerosis model was established in apolipoprotein E null mice (ApoE^-/-^) fed with high fat diet (HFD), and a highly specific small-molecule CDK9 inhibitor, LDC000067, was utilized to treat the atherosclerotic mice. The results demonstrated that treatment with pharmacological CDK9 inhibitor alleviated atherosclerosis by reducing CDK9 phosphorylation and then inhibiting inflammation and phenotypic switching of VSMCs.

## RESULTS

### CDK9 colocalized with VSMCs in atherosclerotic lesions

**Figure 1:**
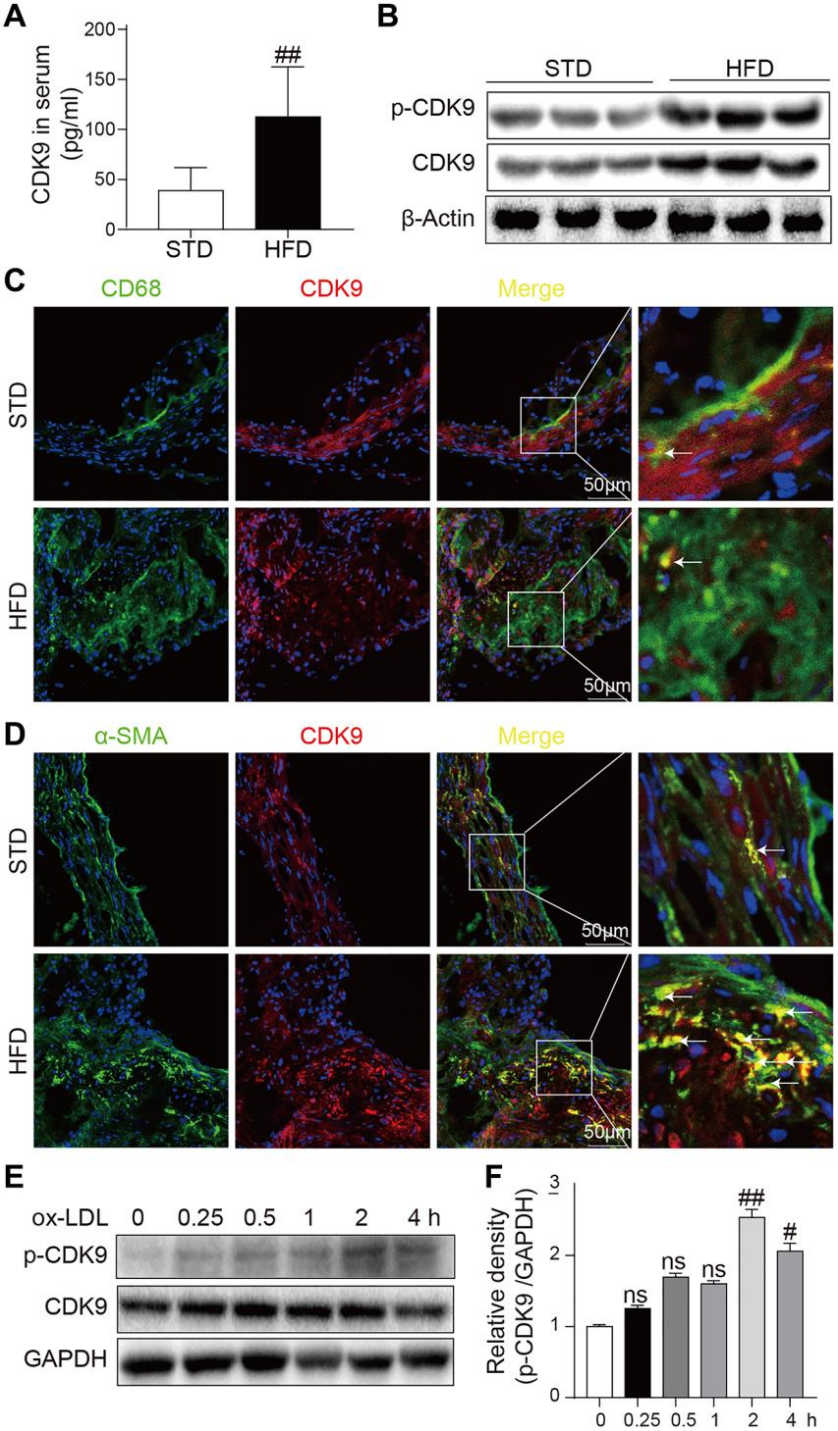
**CDK9 is expressed in VSMCs in atherosclerotic lesions.** (**A**) Serum levels of CDK9 were detected using ELISA assay (*n* = 8; ^**^P < 0.01 compared to STD). (**B**) p-CDK9 and CDK9 protein levels in aortas of ApoE^-/-^ mice were detected by western blotting. (**C**) Representative immunofluorescence staining of CDK9 (red) and macrophage marker CD68 (green). Tissues were counterstained with DAPI (blue). Yellow arrows indicate co-location of CDK9 and CD68 stanning (scale bar = 50 μm). (**D**) Representative immunofluorescence staining of CDK9 (red) and VSMCs marker α-SMA (green). Tissues were counterstained with DAPI (blue). Yellow arrows indicate co-location of CDK9 and α-SMA stanning (scale bar = 50 μm). (**E**, **F**) Western blot analysis of p-CDK9 and CDK9 protein levels in VSMCs challenged with 50 μg/ml ox-LDL for the indicated time points (*n* = 8; ^##^*P* < 0.01 compared to 0h).

The CDK9 level was increased in the serum of patients with atherosclerosis and could be a potential biomarker of atherosclerosis. Therefore, firstly the CDK9 level in the mouse atherosclerosis model was analyzed. The results showed that ApoE^-/-^ mice fed an HFD had increased the serum CDK9 level compared to the mice fed a standard diet (STD) (Figure 1A). Similarly, the levels of CDK9 protein and phosphorylated CDK9 (p-CDK9) in the aortas were significantly higher in the HFD-fed mice (Figure 1B). Further immunofluorescence double-staining showed that CDK9 was colocalized with smooth muscle α-actin (α-SMA), but not macrophage marker CD68 (Figure 1C, 1D). We also see that, although macrophages may express CDK9 in STD mice, only CDK9 in VSMCs was significantly increased in HFD mice. Therefore, we conclude that CDK9 in VSMCs plays an important role in the pathogenesis of atherosclerosis. Therefore, human VSMC cell line was exposed to ox-LDL. Interestingly, the results showed that ox-LDL did not change the CDK9 protein level up to 4 h treatment, but significantly increased p-CDK9 at 1~4 h post exposure (Figure 1E, 1F). The phenomenon that the level of p-CDK9 begin to slightly decrease after ox-LDL treatment for 4h might be due to the consuming or even exhaustion of oxLDL in the cultural medium after 4-hour incubation.

### CDK9 inhibitor treatment reduced atherosclerotic lesions in the ApoE^-/-^ mice fed with HFD

**Figure 2:**
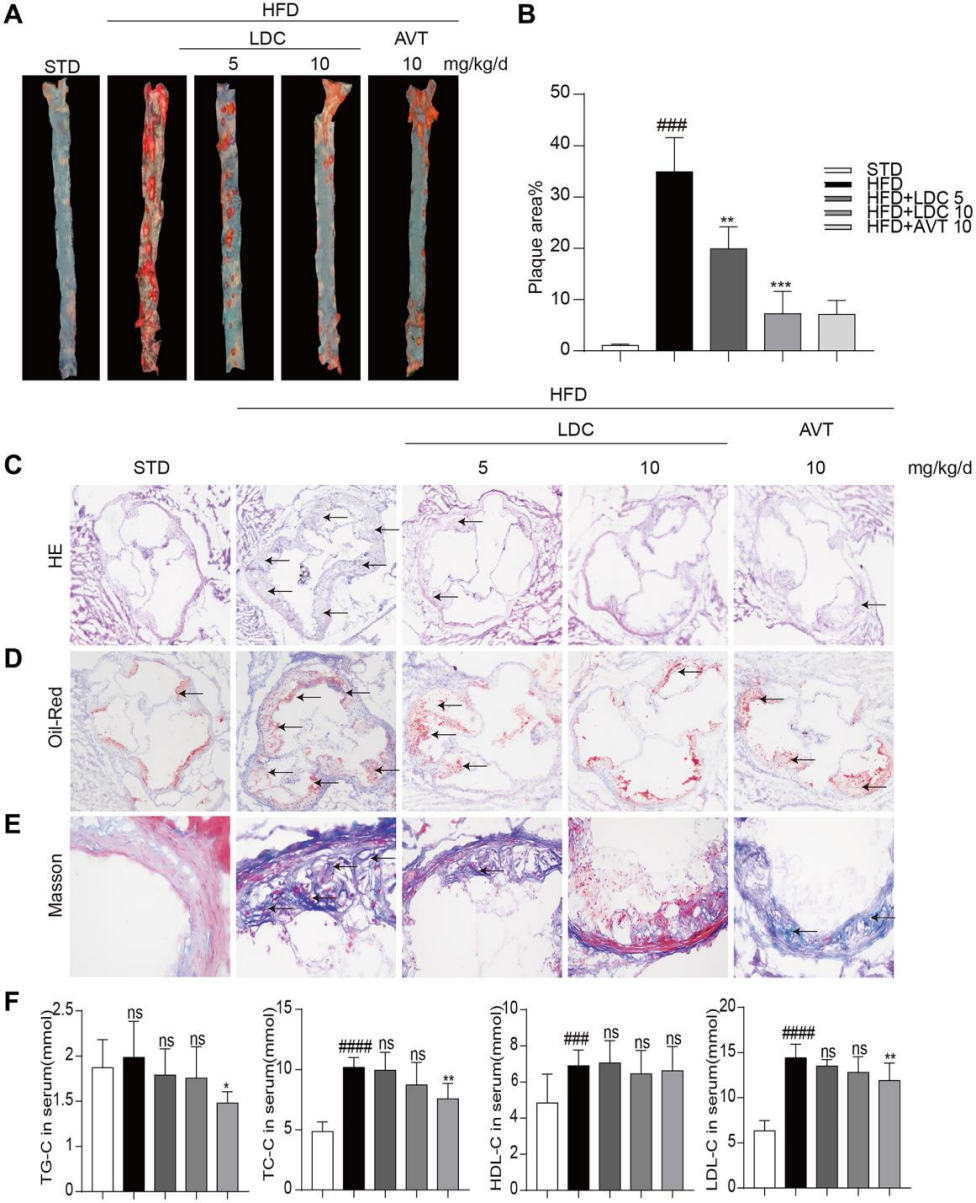
**CDK9 inhibitor reduces indices of atherosclerotic lesions in ApoE^-/-^ mice fed with HFD**. (**A**–**B**) Representative en face Oil Red O staining and quantification of Oil Red O-positive lipid area in the aorta (*n* = 8; ^###^*p* < 0.001 compared to STD, ^***^p < 0.001 compared to HFD;). (**C**) Photomicrographs showing representative H&E staining of atherosclerotic lesions (scale bar = 500 μm). (**D**) Oil Red O staining of atherosclerotic lesions in the aortic root (scale bar = 500 μm) and quantification lesions area highlighted by Oil Red O staining. (**E**) Representative images of Masson’s Trichome staining for collagen deposition (scale bar = 50 μm). (**F**) Serum levels of TG, TC, LDL and HDL (*n* = 8; ^#^*P* < 0.05, ^##^*P* < 0.01 and ^###^*p* < 0.001 compared to STD; ^*^P < 0.05, ^**^P < 0.01 compared to HFD).

Next, LDC000067 was applied to the ApoE^-/-^ mice to explore the potential role of CDK9 in atherosclerosis. HFD feeding significantly increased mouse body weight compared to STD feeding, while LDC000067 treatment had no effect on body weight change (Supplementary Figure 1). Oil Red O staining in the entire aorta showed that the plaque area markedly increased in the HFD-mice compared to the STD-mice, and the increased lesion area were notably reduced by treatment with LDC000067 (both low and high doses) or Atorvastatin (Figure 2A–2B). As depicted in Figure 2C–2D and Supplementary Figure 2A, the plaque areas in the aortic sinus of LDC000067-treated mice and Atorvastatin-treated mice were dramatically smaller than that of the vehicle-treated HFD-fed mice. Masson’s trichome staining showed similar results in reduced collagen deposition (Figure 2E and Supplementary Figure 2B). Serum lipid analysis showed increased total triglycerides (TG), total cholesterol (TC), low-density lipoprotein cholesterol (LDL-C) and high-density lipoprotein cholesterol (HDL-C) after HFD feeding, and LDC000067 had no effect on those HFD-induced lipid changes, while Atorvastatin treatment prevented HFD-induced TG, TC and LDL-C increases (Figure 2F). It is observed that HFD feeding increases serum HDL-cholesterol level in Figure 2F. This result needs further validation, but we may guess that HFD feeding increases the level of cholesterol, which needs more HDL to transfer.

### CDK9 inhibitor prevented HFD-induced inflammation and phenotypic switching of VSMCs *in vivo*

**Figure 3:**
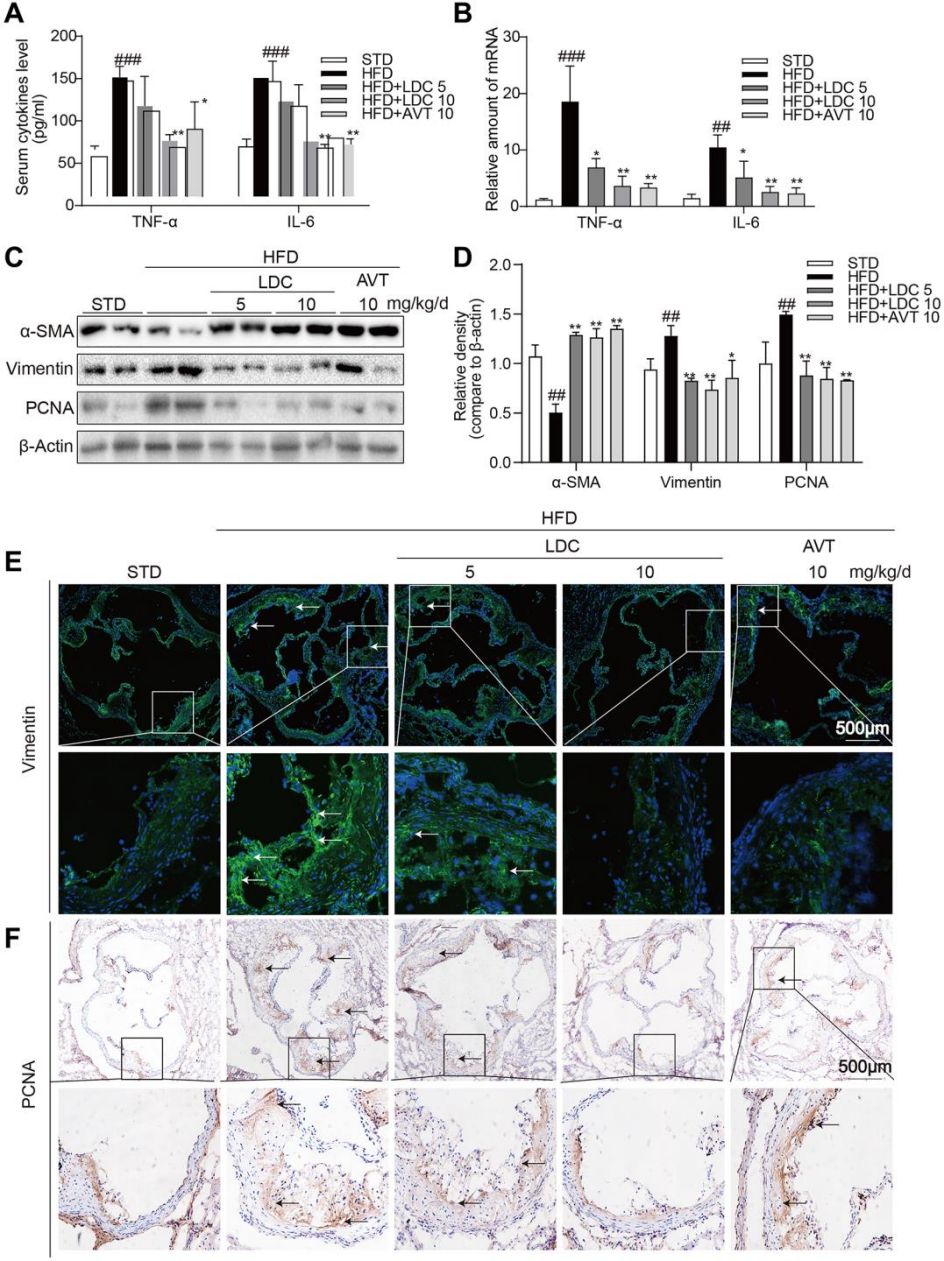
**CDK9 inhibitor reduces HFD-induced inflammation phenotype switch of VSMCs *in vivo.*** (**A**) TNF-α and IL-6 protein levels in the serum were detected using ELISA assay (*n* = 8; ^###^*p* < 0.001 compared to STD; ^*^p < 0.05, ^**^p < 0.01 compared to HFD). (**B**) TNF-α and IL-6 mRNA levels in the aorta were detected using real-time qPCR assay (*n* = 8; ^##^*p* < 0.01, ^###^*p* < 0.001 compared to STD; ^*^p < 0.05, ^**^p < 0.01 compared to HFD). (**C**–**D**) Western blot assay showed the expressions of α-SMA, Vimentin and PCNA in the whole aorta from ApoE^-/-^ fed a normal (STD) or HFD for 16 weeks. Densitometric quantification for blots was shown in panel D (*n* = 8; ^##^*p* < 0.01 compared to STD; ^*^p < 0.05, ^**^p < 0.01 compared to HFD). (**E**) Representative immunofluorescence staining images for Vimentin (green) in aortic roots. Tissues were counterstained with DAPI (blue) (scale bar = 500 μm). (**F**) Representative images of PCNA staining of aortic roots (scale bar = 500 μm; DAB chromogen staining (brown)).

As shown in Figure 3A, HFD-fed ApoE^-/-^ mice showed increased serum levels of inflammatory cytokines including tumor necrosis factor-α (TNF-α) and interleukin-6 (IL-6), and these increases were ameliorated with high-dose LDC000067 and Atorvastatin treatments. Similarly, the increased mRNA levels of these cytokines in the aortas were downregulated with LDC000067 and Atorvastatin treatments (Figure 3B).

Because CDK9 expression in the atherosclerotic lesion colocalized with α-SMA and inflammatory responses often accompanied with phenotypic switching of VSMCs, markers of VSMCs and cell proliferation were determined in the aortas. Western blot showed significant decrease in α-SMA and increases in Vimentin (the marker of synthetic VSMCs) and proliferating cell nuclear antigen (PCNA, a marker of cell proliferation) in HFD-fed ApoE^-/-^ mice, while those changes were all reversed by LDC000067 and Atorvastatin treatments (Figure 3C, 3D). Immune staining further confirmed decreased Vimentin and PCNA in the atherosclerotic lesions of HFD-fed ApoE^-/-^ mice (Figure 3E, 3F).

### CDK9 inhibitor or CDK9 siRNA prevented ox-LDL-induced inflammation and phenotypic switching of VSMCs *in vitro*

**Figure 4:**
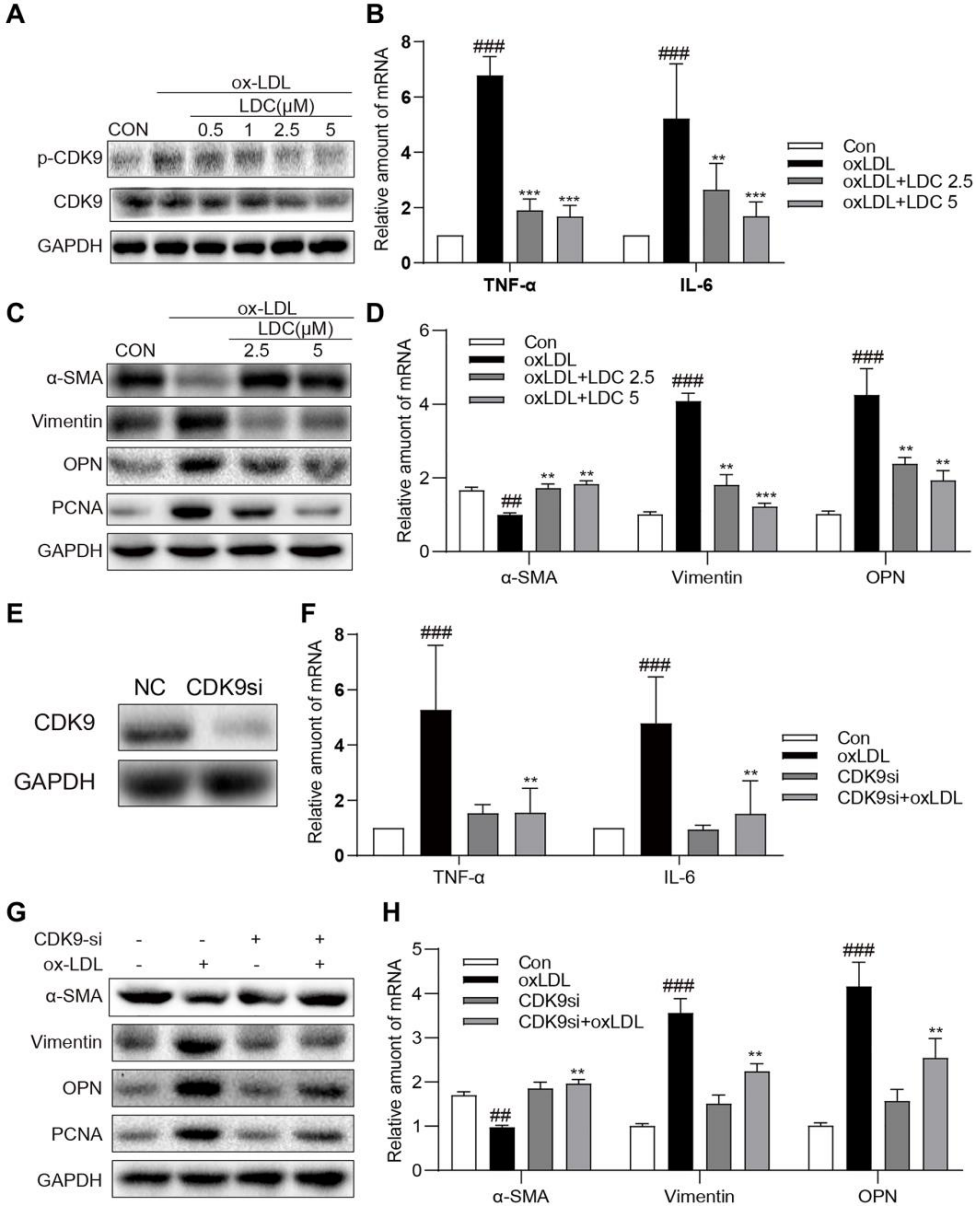
**Inactivation of p-CDK9 by CDK9 inhibitor or CDK9-si prevented ox-LDL-induced inflammation and phenotype switch of VSMCs *in vitro***. (**A**) LDC000067 suppressed ox-LDL-induced activation of p-CDK9. VSMCs were pretreated with LDC000067 (indicated concentrations) for 1 h and then incubated with ox-LDL (50 μg/mL) for 2 h. The levels of *p*-CDK9 and CDK9 were detected by western blot. (**B**–**D**) VSMCs were treated with LDC000067 (2.5 or 5 μM) for 1 hour and then exposed to ox-LDL (50 μg/mL) for 6 h (in panels **B**), 24 h (in panels **C**) or 12 h ((in panels **D**). (**B**) The levels of TNF-α and IL-6 were detected using real-time qPCR assay. (**C**) Expressions of α-SMA, Vimentin, OPN and PCNA in the cultural medium were detected by western blot. (**D**) The mRNA levels of α-SMA, Vimentin, OPN were detected using real-time qPCR assay (*n* = 3 ^##^*P* < 0.01, ^###^*P* < 0.001, compared to control; ^*^P < 0.05, ^**^P < 0.01, ^***^P < 0.001, compared to ox-LDL). (**E**–**H**) VSMCs were transfected with siRNA against CDK9 and then incubated with ox-LDL (50 μg/mL) for 6 h (in panels **G**), 24 h (in panels **H**) or 12 h ((in panels **H**). (**E**) VSMCs were transfected with siRNA against CDK9 for 6 h and then detected expression of CDK9 by western blot. (**F**) The level of TNF-α and IL-6 were detected using real-time qPCR assay. (**G**) Expressions of α-SMA, Vimentin, OPN and PCNA in the cultural medium were detected by western blot. (**H**) The mRNA level of α-SMA, Vimentin, OPN were detected using real-time qPCR assay (*n* = 3; ^##^*P* < 0.01, ^###^*P* < 0.001 compared to control; ^*^P < 0.05, ^**^P < 0.01, compared to ox-LDL).

To further confirm the *in vivo* effects of CDK9 inhibition on inflammation and phenotypic switching of VSMCs, CDK9 inhibitor and CDK9 siRNA were applied to ox-LDL-treated human VSMCs. First of all, MTT assay showed no significant impact of LDC000067 on cell viability at up to 5 μM (Supplementary Figure 3). Figure 4A showed that, following treatment with LDC000067 for 2 h, ox-LDL-induced p-CDK9 was obviously downregulated at 2.5 and 5 μM, which were used in further *in vitro* experiments (Figure 4A and Supplementary Figure 4).

Firstly, VSMCs were treated with ox-LDL with or without LDC000067, and the mRNA levels of TNF-α and IL-6 were determined by real-time qPCR. As expected, the ox-LDL-increased mRNA levels of TNF-α and IL-6 in VMSCs were prevented by LDC000067 treatments at 2.5 and 5 μM (Figure 4B). Secondly, as shown in Figure 4C–4D, pretreatment with LDC000067 suppressed both protein and mRNA levels of Vimentin, osteopontin (OPN), and PCNA, and rescued the expression of α-SMA induced by ox-LDL (Figure 4C and Supplementary Figure 5). These results of LDC000067 treatment were further confirmed by utilizing CDK9-targeting siRNA (Figure 4E and Supplementary Figure 6), i.e., CDK9 gene knockdown significantly blocked ox-LDL-induced inflammatory response, proliferation and phenotypic switching of VSMCs (Figure 4F–4H and Supplementary Figure 7).

### CDK9 inhibition or knockdown reduced ox-LDL-induced inflammatory responses and phenotypic switching of VSMCs by suppressing NF-κB pathway

**Figure 5:**
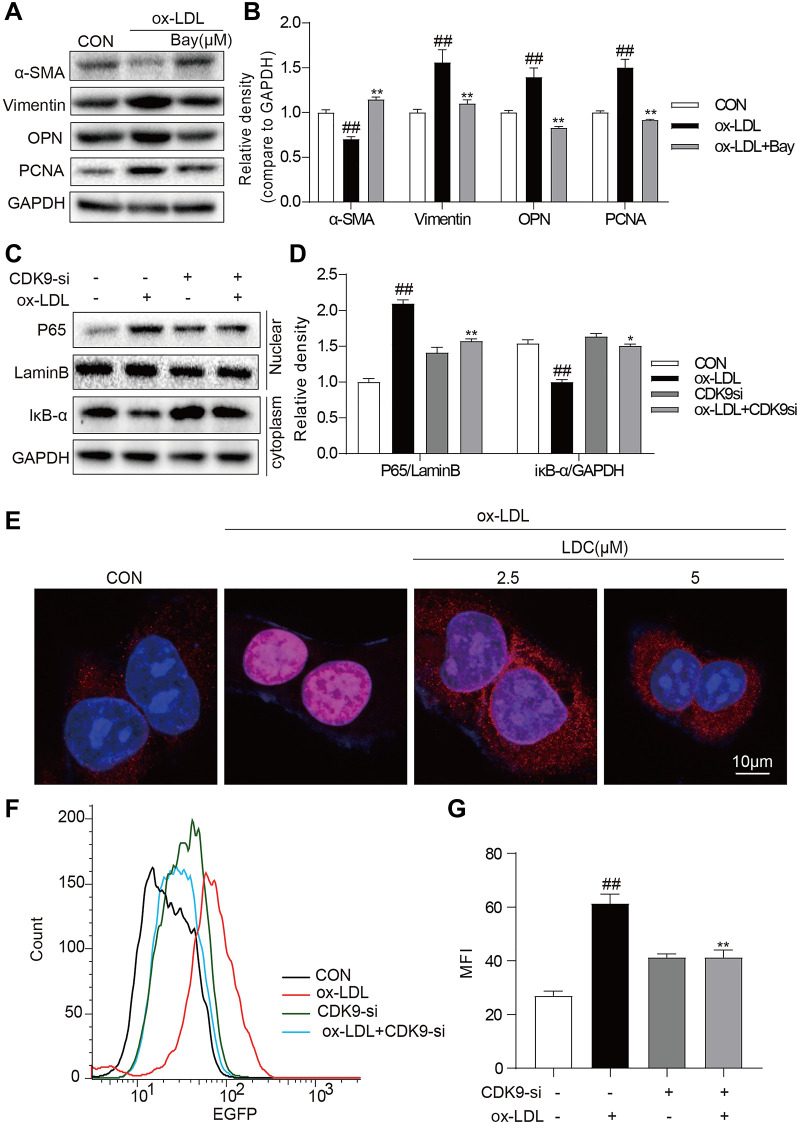
**CDK9 inhibitor reduces inflammatory responses and phenotype switch of VSMCs exposed to ox-LDL by suppressing NF-κB pathway.** (**A**–**B**) VSMCs were treated with the Bay (10μM) for 1 h, and then stimulated with ox-LDL (50μg/mL) for 24 h. The expressions of α-SMA, Vimentin, and PCNA were detected by western blot ((*n* = 3; ^##^*P* < 0.01, compared to control; ^*^P < 0.05, ^**^P < 0.01, compared to ox-LDL). (**C**–**D**) transfected with siRNA against CDK9 and then incubated with ox-LDL (50 μg/mL) for 1 h. The IκB-α protein level in cell lysate was detected by western blot; and the nuclear fraction was isolated and the nuclear level of NF-κB p65 was measured by western blot (*n* = 3; ^##^*P* < 0.01, compared to control; ^*^P < 0.05, ^**^P < 0.01 compared to ox-LDL). (**E**) VSMCs were pretreated with LDC000067 (2.5 or 5 μM) for 1 h, and then stimulated with ox-LDL (50 μg/mL) for 1 h. Nuclear translocation of NF-κB p65 was measured by immunofluorescence staining (scale bar = 10 μm). (**F**–**G**) NF-κB-RE-EGFP reporter VSMCs were transfected with siRNA against CDK9 and then stimulated with ox-LDL (50 μg/mL) for 6 h. NF-κB activity is shown as mean fluorescence intensity (MFI) in flow cytometry histogram (n = 3; ^##^*P* < 0.01, compared to control; ^**^P < 0.01, compared to ox-LDL).

It is well known that NF-κB signal pathway plays key roles in inflammation and mediates the development of atherosclerosis. Here an NF-κB specific inhibitor BAY 11–7082 (BAY) could reverse ox-LDL-induced inflammation and phenotypic switching of VSMCs (Figure 5A, 5B). The translocation of NF-κB subunit p65 from the cytoplasm to the nuclear to initiate the gene expression of inflammatory cytokines is a hallmark event in NF-κB activation. Then, we evaluated the effect of CDK9 inhibitor against ox-LDL-induced NF-κB activation via examining p65 translocation in VSMCs. Western blot showed that CDK9 knockdown by siRNA greatly inhibited ox-LDL-induced nuclear translocation of NF-κB p65 in VSMCs (Figure 5C, 5D). Immunofluorescence staining analysis showed that ox-LDL increased nuclear p65 level, while treatment with LDC000067 dose-dependently reduced p65 nuclear translocation, indicating that LDC000067 inhibited ox-LDL-induced NF-κB activation (Figure 5E). Furthermore, a stable VSMC line expressing NF-κB activity reporter (EGFP) was generated and exposed to ox-LDL. As shown in Figure 5F and 5G, CDK9 knockdown by siRNA decreased ox-LDL-induced NF-κB activity.

## DISCUSSION

CDK9 is responsible for cell cycle control and progression in a lot of cell types [16]. In this study, inflammation and phenotypic switching of VSMCs were observed in HFD-induced atherosclerosis in ApoE^-/-^ mice, which were accompanied with increased CDK9 in the serum and atherosclerotic lesions where it colocalized with VSMCs. CDK9 inhibitor LDC000067 treatment significantly suppressed HFD-induced inflammation, cell proliferation and phenotypic switching of VSMCs resulting in reduced atherosclerosis in the ApoE^-/-^ mice, which were further confirmed *in vitro* using ox-LDL-treated human VSMCs. In addition, CDK9 inhibition does not affect HFD-induced serum lipid profile, indicating that CDK9 is not involved in the pathways of lipid uptake and metabolism. Further *in vitro* studies with LDC000067 and CDK9 siRNA demonstrated that CDK9 mediated ox-LDL-induced inflammation and phenotypic switching of VSMCs via NF-κB signaling pathway.

It is well known that inflammation is an active driver of atherosclerotic plaque development and a risk factor for atherosclerotic events [3, 17]. Han et al. have reported that CDK9 was highly elevated in the serum of patients with atherosclerosis and could be a potential biomarker of atherosclerosis [15]. Many studies have demonstrated that CDK9 is actively involved in many inflammatory diseases [18, 19], and the Thr186 site of CDK9 is sensitive to phosphorylation, which initiates downstream responses [20, 21]. In this study, an interesting result found in Figure 1 is that both CDK9 and p-CDK9 in the aortas of HFD mice increase significantly, while only p-CDK9 was increased in the ox-LDL-challenged VSMCs. In animal experiments, the development of atherosclerosis was accompanied by the proliferation of VSMCs, and the increase in the number of VSMCs leads to the increase in CDK9 protein level. *In vitro*, however, a short time stimulation by ox-LDL failed to increase the number of VSMCs, therefore, only increased p-CDK9 was detected in the ox-LDL-stimulated VSMCs. In addition, CDK9 activation and inflammatory status may induce its overexpression in a positive feedback in long-term HFD feeding mice. As expected, CDK9 specific inhibitor LDC000067 treatment significantly downregulated inflammatory cytokines including TNF-α and IL-6 and attenuated atherosclerotic plaque development HFD-fed ApoE^-/-^ mice.

Previous studies have shown that the role of vascular smooth muscle in the progression of atherosclerosis has been greatly underestimated [22]. In addition to inflammation, phenotypic switching of VSMCs also plays an important role in the pathological process of atherosclerosis [23–25]. VSMCs can switch its phenotype from contractile to synthetic in response to certain stimuli [26]. In a stable state, mature VSMCs stably express contractile phenotypic markers, such as α-SMA, SM22a, SM-myosin heavy chain, and H1-calponin. Once stimulated, VSMCs change to a synthetic type and upregulated synthetic markers including Vimentin and OPN [27]. In this study, LDC000067 treatment decreased synthetic markers and increased contractile markers *in vitro* and *in vivo*. It was further confirmed by CDK9 knockdown using siRNA that also inhibited ox-LDL-induced phenotypic switching of VSMCs from a contractile phenotype to a synthetic phenotype. These results indicate a role of p-CDK9-mediated phenotypic switching of VSMCs in atherosclerosis. Previous research found that CDK9 inhibitor Flavopiridol attenuated leukocyte-endothelial interaction and then suppressed inflammation [28]. Although we did not examine the expression level and co-localization of CDK9 in the arterial endothelium, Figure 1C and 1D indicated that CDK9 is mainly expressed in cells in the middle layer of the aorta, rather than the inner layer. This result means CDK9 may be not significantly expressed in vascular endothelial cells located in the inner layer of aorta, at least in atherosclerosis. CDK9 is a multifunctional kinase in many cell types. Whether CDK9 regulating leukocyte-endothelial interaction also has an important role in atherosclerosis needs to be further investigated in the future.

NF-κB activation is a key signaling of inflammation and is close association with the progress of atherosclerosis [29]. Ox-LDL will strongly activate NF-κB signal via binding its membrane surface receptor LOX-1 and then induce proinflammatory cytokine expression such as TNF-α and IL-6 [9]. The increased expression of proinflammatory cytokines will then trigger the phenotypic switching of VSMCs from a contractile phenotype to a synthetic phenotype accompanied with the loss of expression of smooth muscle contractile proteins and induction of proliferation, and ultimately result in the development of atherosclerosis. [8] In this study, the role of NF-κB in ox-LDL-induced inflammation and phenotypic switching of VSMCs was confirmed by the inhibitor BAY. Interestingly, as demonstrated in Figure 5, CDK9 inhibitor LDC000067 had similar inhibitive effect on the NF-κB signaling. These results indicate that CDK9 inhibitor reduced ox-LDL-induced inflammatory responses and phenotypic switching of VSMCs by suppressing NF-κB pathway and then improved atherosclerosis. Studies have shown that CDK9 plays a role in inflammatory responses through the activation of mitogen-activated protein kinase (MAPK) pathways [30]. Our previous study also showed that CDK9 could activate MAPKs in renal cells [31]. Considering that NF-κB is a downstream transcriptional factor of MAPKs, we may speculate that CDK9 up-regulates NF-κB activity via activating MAPKs. In addition, previous studies have shown that knockout of KLF4 alleviates the development of atherosclerosis by inhibiting the phenotypic switching of vascular smooth muscle [22, 32, 33]. Besides, Ye Ding et al. have reported that KLF4’s upstream promoter contains a putative NF-κB p65 binding site [5]. Therefore, we speculated that KLF4 may mediate in the regulation of phenotypic switching by CDK9 through the NF-κB pathway.

## CONCLUSION

In conclusion, CDK9 was increased in the serum of HFD-fed ApoE^-/-^ mice and its expression in atherosclerotic lesions colocalized with VSMCs. Treatment with CDK9 inhibitor LDC000067 dramatically reduced hyperlipidemia-induced inflammation and phenotypic switching of VSMCs and ultimately prevented atherosclerosis progress without changing serum lipid levels, indicating that CDK9 may be a new target for the prevention of atherosclerosis. However, one potential concern regarding the clinical use of LDC000067 currently may be the narrow therapeutic window and potential adverse effects. We observed that LDC000067 at 10 μM induced cell death of VMSCs (Supplementary Figure 3). However, the promising results obtained in our study warrant further investigation. Medicinal chemists should start to modify LDC000067 to explore new CDK9 inhibitors with low toxicity for the treatment of atherosclerosis.

## METHODS

### Reagents and chemicals

The CDK9 inhibitor, LDC000067, was purchased from Selleck (Houston, TX). LDC000067 was dissolved in dimethyl sulfoxide (DMSO) for *in vitro* experiments and in CMC-Na (1%) for *in vivo* study. Atorvastatin was purchased from Pfizer Ireland Pharmaceuticals (New York, USA). Antibodies against NF-κB p65 (sc-8008), Lamin B (sc-56144) and GAPDH (sc-32233) were from Santa Cruz Biotechnology (Santa Cruz, CA, USA); antibodies against CDK9 (2316S), p-CDK9 (2549S) and IκB (4814S) were from Cell Signaling Technology (Danvers, MA); antibodies against α-SMA (ab32575), Vimentin (ab8978), OPN (ab8448), PCNA (ab29), β-actin (ab8227) and CD68 (ab955) were from Abcam (Cambridge, MA, USA). Mouse CDK9 ELISA kit (E-EL-M0378c) was from Elabscience Biotechnology Co., Ltd (Wuhan, China).

### Cell culture

Human VSMC cell line was purchased from Shanghai Yichen Biotechnology co., LTD (Shanghai, China). VSMCs were cultured at 37°C with 5% CO_2_ in Ham's F-12K (Kaighn's) Medium (Gibco/BRL life Technologies, Eggenstein, Germany) containing 10% fetal bovine serum (Hyclone, Logan, UT), 100 U/ml penicillin, and 100 mg/mL streptomycin. VSMCs were treated with Ox-LDL (Peking Union Biology Company, Beijing, China) at 50 μg/mL as used in previous study [34].

### Cell viability assay

VSMCs were seeded into a 96-well plate at 5 × 10^4^ cells per well. Cells were allowed to attach to the wells before treatment with LDC000067 for 24 h at various concentrations. Cells were incubated with MTT reagents for 4 h and measured at 490 nm. Cell viability was defined as the percentage of absorbance compared to the control.

### siRNA-induced gene silencing

CDK9 siRNA (5′-GCUGCUAAUGUGCUUAUCA-3′) was purchased from Gene Pharma Co., LTD (Shanghai, China), and transiently transfected with Lipofectamine^®^ 2000 (Invitrogen, Carlsbad, California).

### Generation of stable NF-κB EGFP VSMCs

VSMCs were transfected with p-LV-NF-κB-RE-EGFP lentiviral particles to generate the stable cell line containing NF-κB response element-driven EGFP reporter. Briefly, HEK293T cells were co-transfected with lentiviral plasmid (p-LV-NFκB-RE-EGFP) and packaging plasmids (psPAX2 and pMD2.G) using PEI (Sigma-Aldrich). The supernatant was collected at 48 h post transfection and filtered using a 0.45 μm filter, and then the supernatant was added to VSMCs with 8 μg/mL polybrene (Sigma, H9268) for 12 h. VSMCs were selected with 2 μg/mL puromycin (MCE, HY-B1743A). VSMCs were challenged with 0.5 μg/mL lipopolysaccharide (LPS; Sigma-Aldrich) for 6 h, and cell clones compared to LPS-free control showing at least two-fold increase in fluorescence were considered stable NF-κB-EGFP expressing cells.

### Animal study

All experimental procedures and animal care followed The Detailed Rules and Regulations of Medical Animal Experiments Administration and Implementation, Ministry of Public Health, P.R. China. All animal study protocols were approved by Wenzhou Medical University Animal Policy and Welfare Committee and adhered to the NIH guidelines (Guide for the care and use of laboratory animals). Fifty 8-week-old male ApoE^-/-^ mice were purchased from HFK Bioscience Co. Ltd (Beijing, China) and randomly divided into five groups (*n* = 10 for each group), i.e., one control group fed with standard diet (STD) containing 10 kcal.% fat, 20 kcal.% protein and 70 kcal.% carbohydrate (HFK Bioscience Co. Ltd, Beijing, China) and four HFD groups fed with containing 60 kcal.% fat, 20 kcal.% protein and 20 kcal.% carbohydrate (HFK Bioscience Co. Ltd) [35] for 16 weeks. On the 9th week, the four HFD-fed groups were treated with vehicle control, positive control (Atorvastatin, 10mg/kg/day), low- and high-dose of LDC000067 (5 and 10mg/kg/day, respectively). The used doses of LDC000067 and Atorvastatin were decided according to previous studies [12, 14, 36]. The HFD vehicle control and STD groups received 1% CMC-Na solution. Body weights were recorded weekly. After 12 weeks of treatment, the mice were sacrificed and the tissues were collected and immediately frozen.

### Atherosclerotic lesion analysis

Whole aorta was excised, opened longitudinally from heart to the iliac arteries, and stained with Oil Red O for analysis of lesions. The heart and proximal aorta were removed and embedded in optimum cutting temperature compound. Serial 5 μm-thick cryosections from the middle portion of the ventricle to the aortic arch were collected. Sections were stained with oil red O, H&E, and Masson trichrome staining for the analysis and characterization of atherosclerotic lesions in aortic sinus.

### Immunohistochemistry

Paraffin sections were deparaffinized and rehydration. After antigen retrieval, slides were incubated with 3% H_2_O_2_ for 10 min to block endogenous peroxidase activity and were blocked with 1% bovine serum albumin for 30 min. Then slides were incubated overnight at 4°C with PCNA (1:200) and TNF-α antibody (1:200). Horseradish peroxidase-conjugated secondary antibody (Santa Cruz; 1:500) and DAB were used for detection.

### Immunofluorescence assay

Frozen sections were used for Vimentin co-staining of CD68/CDK9 and α-SMA/CDK9. Slides were fixed in cold methanol, permeabilized with 0.3% Triton-X, blocked with 5% bovine serum albumin for 30 min, and then incubated overnight with primary antibodies (1:200). Alexa-488/561 conjugated secondary antibodies (Abcam, 1:500) were used for detection. Slides were counterstained with DAPI. For NF-κB p65 immunofluorescence staining in VSMCs, cells were fixed with 4% paraformaldehyde and permeabilized with 0.3% Triton X-100. After then, VSMCs were incubated with anti-p65 antibody (1:200) at 4°C overnight. Alexa-488 conjugated secondary antibodies (1:500) was used for detection. Nuclei were stained with the DAPI at room temperature.

### Real-time quantitative PCR

Cells or aortic tissues were homogenized in TRIZOL (Invitrogen, Carlsbad, CA) for total RNA extraction according to the manufacture's protocol. Reverse transcription was performed using PrimeScript RT Reagent Kit with gDNA Eraser (TAKARA, Japan). Quantitative PCR was conducted using iQ™ SYBR Green Supermix (Bio-Rad, Shanghai, China) in QuantStudio™ 3 Real-Time PCR System (Thermo Fisher). Primers targeting genes including TNF-α, IL-6 and β-actin were synthesized by Invitrogen (Invitrogen, Shanghai, China). Primer sequences are listed in the Supplementary Table 1. The mRNA level of each gene was normalized by the corresponding β-actin mRNA level.

### Western blotting assay

Lysates from cells or aortic tissues were separated by 10% sodium dodecyl sulfate-polyacrylamide gel electrophoresis (SDS-PAGE) and electro-transferred to a polyvinylidene fluoride membrane (Bio-Rad Laboratory, Hercules, CA). After blocking (5% non-fat milk in tris-buffered saline containing 0.05% Tween 20, TBS-T) for 1.5 h at room temperature, membranes were incubated with specific antibodies. Immunoreactive bands were detected by incubating with horseradish peroxidase-labelled secondary antibody for 2 h at room temperature and visualizing using enhanced chemiluminescence reagents (Bio-Rad, Hercules, CA, USA).

### Statistical analysis

Data *in vitro* represented at least 3 independent experiments and were expressed as means ± SEM. The statistical significance of differences between groups was obtained by the student’s *t* test or ANOVA multiple comparisons with GraphPad Pro 7.00 (GraphPad, San Diego, CA). *P* value <0.05 was considered significant.

### Availability of data and material

Supplementary information includes 1 Table and 3 Figures. All the other data are available from the authors on request.

## Supplementary Materials

Supplementary Figures

Supplementary Table 1
