# Correction for: Inhibition of CDK9 attenuates atherosclerosis by inhibiting inflammation and phenotypic switching of vascular smooth muscle cells

**DOI:** 10.18632/aging.204017

**Published:** 2022-04-14

**Authors:** Shushi Huang, Wu Luo, Gaojun Wu, Qirui Shen, Zaishou Zhuang, Daona Yang, Jinfu Qian, Xiang Hu, Yan Cai, Nipon Chattipakorn, Weijian Huang, Guang Liang

**Affiliations:** 1 Chemical Biology Research Center, School of Pharmaceutical Sciences, Wenzhou Medical University, Wenzhou, Zhejiang 325035, China; 2 Department of Cardiology, The First Affiliated Hospital, Wenzhou Medical University, Wenzhou, Zhejiang 325035, China; 3 Affiliated Cangnan Hospital, Wenzhou Medical University, Cangnan, Zhejiang, 325000, China; 4 Department of Endocrinology, The First Affiliated Hospital, Wenzhou Medical University, Wenzhou, Zhejiang 325035, China; 5 Cardiac Electrophysiology Research and Training Center, Faculty of Medicine, Chiang Mai University, Chiang Mai, 50200, Thailand; 6 School of Pharmaceutical Sciences, Hangzhou Medical College, Hangzhou, Zhejiang 311399, China

**Keywords:** atherosclerosis, CDK9, pharmacological inhibition, inflammation, phenotypic switching, vascular smooth muscle cells

Original article: Aging. 2021; 13:14892–14909. https://doi.org/10.18632/aging.202998

**This article has been corrected:** The authors replaced Masson’s Trichrome-stained images showing collagen deposition in the HFD and HFD+LDC-5mg groups in **Figure 2E**. Originally, they incorrectly placed there two photos of the same specimen from the HFD+LDC-5mg group with different magnifications. The replacement was done with representative images from the original sets of experiments. These alterations do not affect the results or conclusions of this work.

The correct **Figure 2** is presented below.

**Figure 2:**
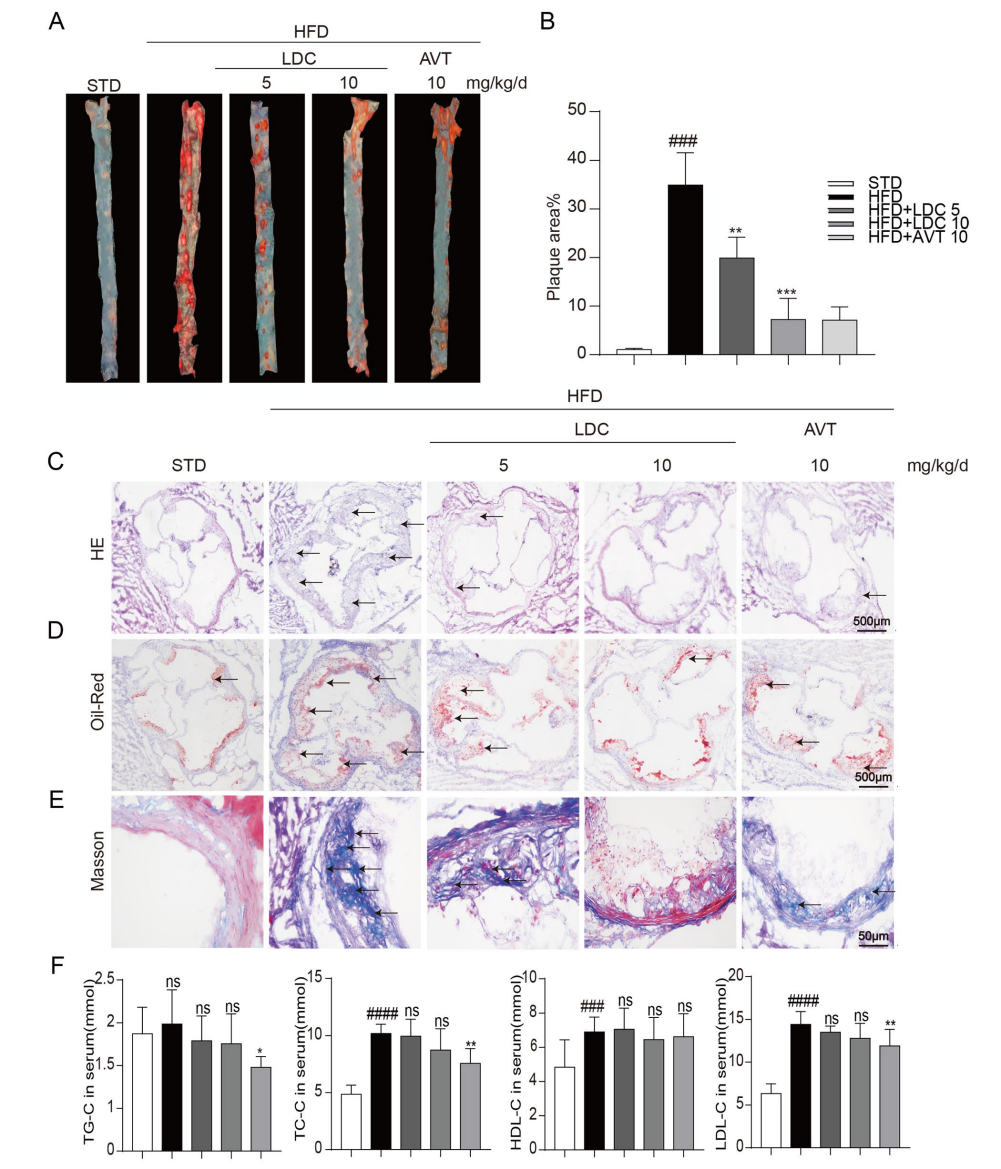
**CDK9 inhibitor reduces indices of atherosclerotic lesions in ApoE^-/-^ mice fed with HFD**. (**A**–**B**) Representative en face Oil Red O staining and quantification of Oil Red O-positive lipid area in the aorta (*n* = 8; ^###^*p* < 0.001 compared to STD, ***p < 0.001 compared to HFD;). (**C**) Photomicrographs showing representative H&E staining of atherosclerotic lesions (scale bar = 500 μm). (**D**) Oil Red O staining of atherosclerotic lesions in the aortic root (scale bar = 500 μm) and quantification lesions area highlighted by Oil Red O staining. (**E**) Representative images of Masson’s Trichome staining for collagen deposition (scale bar = 50 μm). (**F**) Serum levels of TG, TC, LDL and HDL (*n* = 8; ^#^*P* < 0.05, ^##^*P* < 0.01 and ^###^*p* < 0.001 compared to STD; *P < 0.05, **P < 0.01 compared to HFD).

