# Supplementary material for: Inhibition of CDK9 attenuates atherosclerosis by inhibiting inflammation and phenotypic switching of vascular smooth muscle cells
Source: Aging (Albany NY). 2021 Jun 8;13(11):14892–909. doi: 10.18632/aging.202998 (PMC8221363; doi:10.18632/aging.202998)
Supplement: Supplementary Figures [file aging-13-202998-s001.pdf]

SUPPLEMENTARY FIGURES

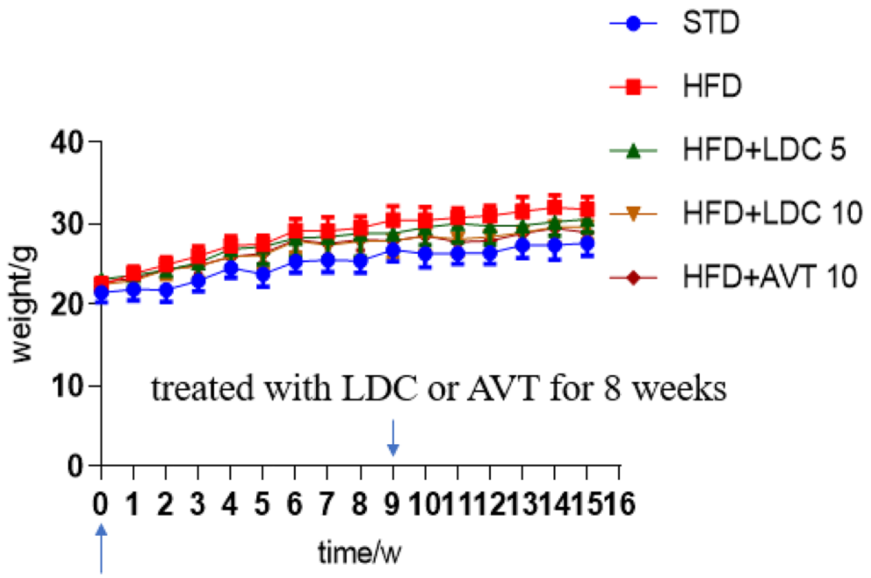

Supplementary Figure 1. Bodyweight change after treatments. $ApoE^{-/-}$ mice were randomly divided into five groups. Four groups were fed with HFD for 16 weeks, while one group was fed with standard diet for 16 weeks that serves as the normal control. On the 9th week, HFD-induced mice were treated with CMC-Na, LDC000067 (LDC, 5 mg/kg/day or 10mg/kg/day) or Atorvastatin (10 mg/kg/day) for 8 weeks (from 9th week to 16th week) by oral gavage. The bodyweights of all mice were measured once per week during the *in vivo* study.

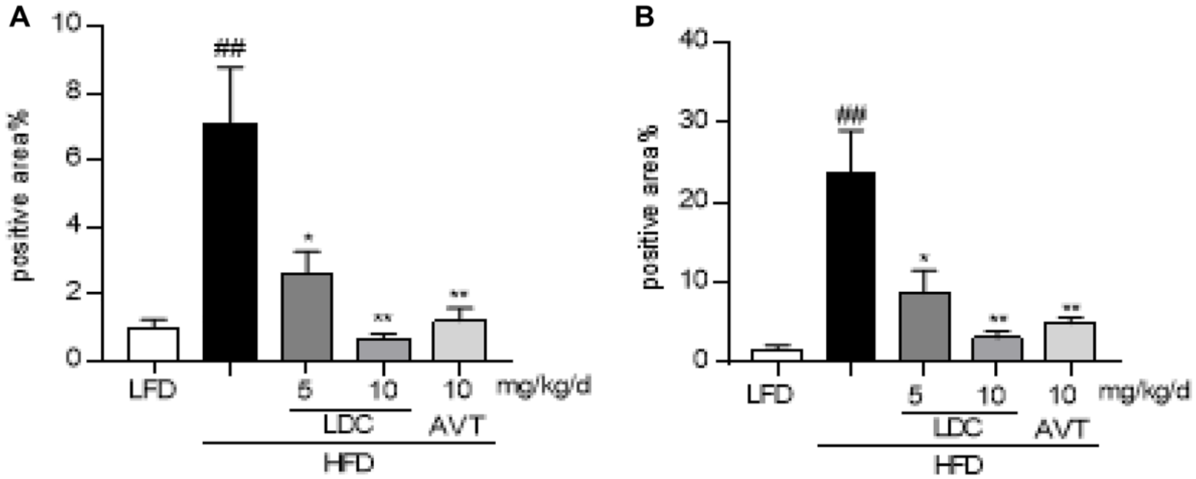

Supplementary Figure 2. Quantification for staining results in Figure 2. (**A**) Quantification for staining results in Figure 2D ($n = 8$, $^{\#\#}P < 0.01$, compared to LFD group; $^{*}P < 0.05$, $^{**}P < 0.01$, compared to HFD group). (**B**) Quantification for staining results in Figure 2E ($n = 8$, $^{\#\#}P < 0.01$, compared to LFD group; $^{*}P < 0.05$, $^{**}P < 0.01$, compared to HFD group).

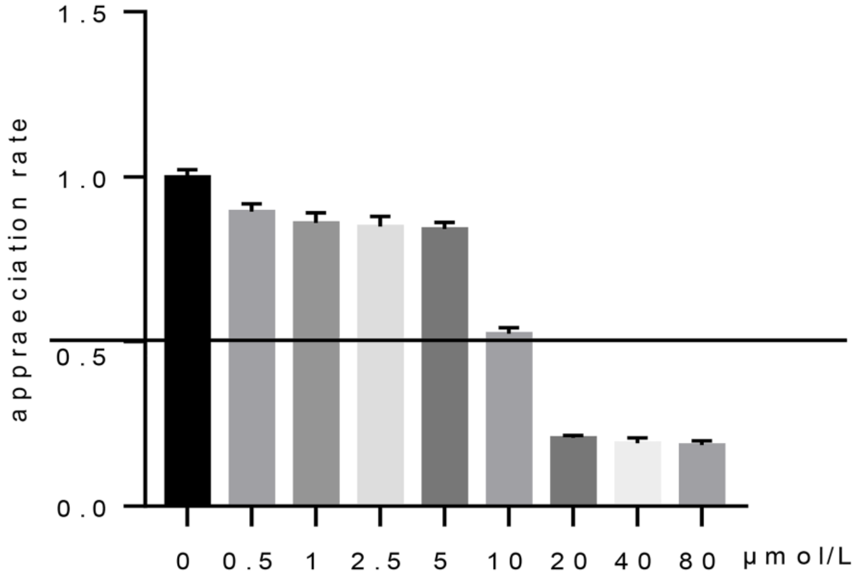

Supplementary Figure 3. Cell viability assay using MTT. VSMCs were allowed to attach before exposure to LDC000067 at increasing concentrations for 24 h. Cells were incubated with MTT reagent for 4 h. Absorbance at 490 nm was measured.

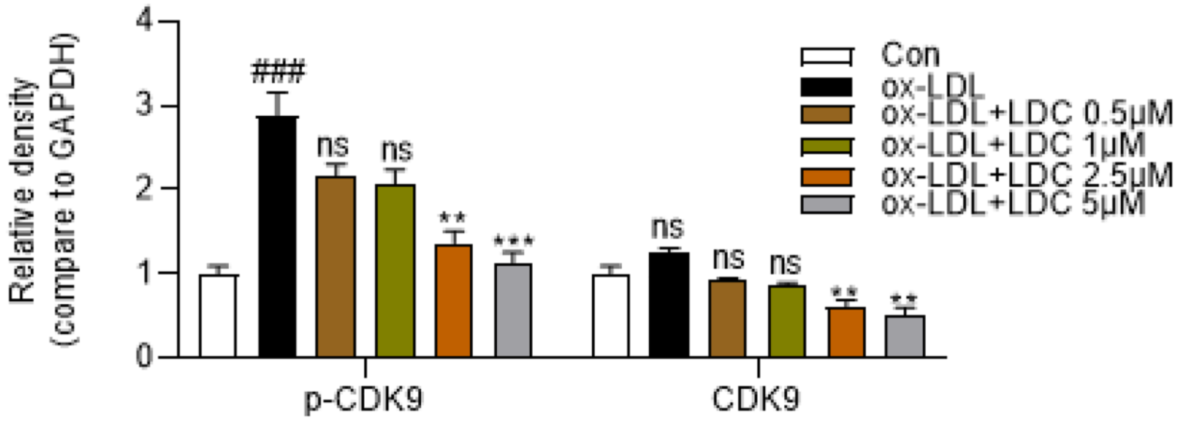

Supplementary Figure 4. Quantification for Figure 4A. VSMCs were pretreated with LDC000067 (indicated concentrations) for 1 h and then incubated with ox-LDL (50 μg/mL) for 2 h. The levels of p-CDK9 and CDK9 were detected by western blot (n = 3; $^{###}P < 0.001$, compared to control; $^{**}P < 0.01$, $^{***}P < 0.001$, compared to ox-LDL).

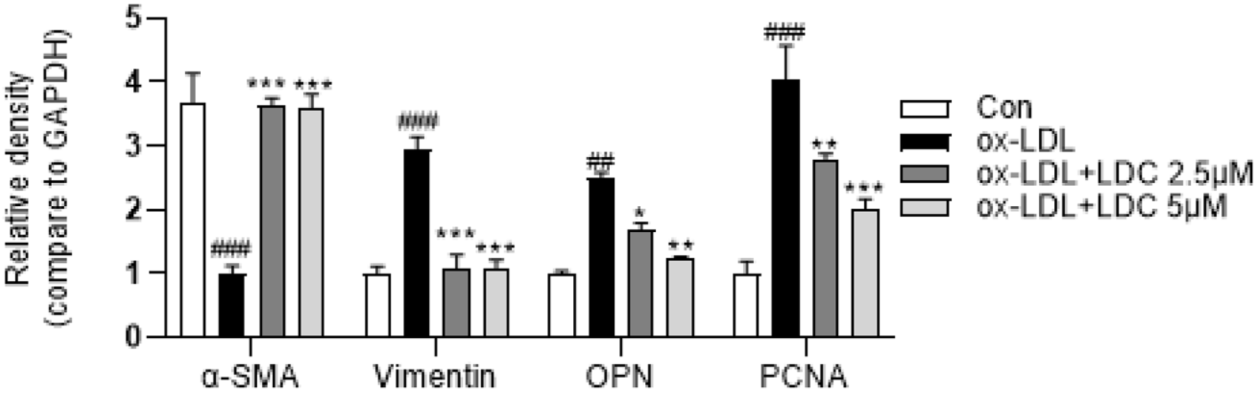

Supplementary Figure 5. Quantification for Figure 4C. VSMCs were treated with LDC000067 (2.5 or 5 μM) for 1 hour and then exposed to ox-LDL (50 μg/mL) for 24 h. Expressions of α-SMA, Vimentin, OPN and PCNA in the cultural medium were detected by western blot (n = 3; $^{##}P < 0.01$, $^{###}P < 0.001$, compared to control; $^{*}P < 0.05$, $^{**}P < 0.01$, $^{***}P < 0.001$, compared to ox-LDL).

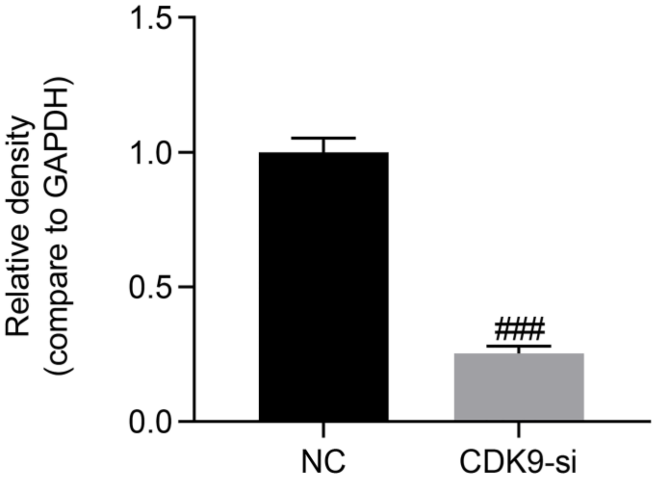

Supplementary Figure 6. Quantification for Figure 4E. VSMCs were transfected with siRNA against CDK9 for 6 h and then detected expression of CDK9 by western blot (n = 3; $^{\#\#}P < 0.01$, compared to NC).

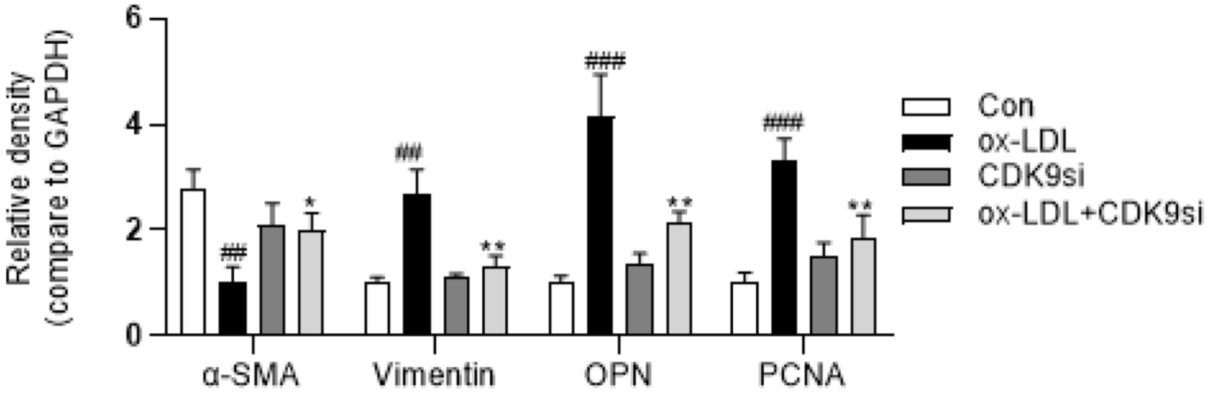

Supplementary Figure 7. Quantification for Figure 4G. VSMCs were transfected with siRNA against CDK9 and then incubated with ox-LDL (50 μg/mL) for 24 h. Expressions of α-SMA, Vimentin, OPN and PCNA in the cultural medium were detected by western blot (n = 3; $^{\#\#}P$ < 0.01, $^{\#\#\#}P$ < 0.001 compared to control; $^{*}P$ < 0.05, $^{**}P$ < 0.01, compared to ox-LDL).
