# Supplementary material for: Inhibition of CDK9 attenuates atherosclerosis by inhibiting inflammation and phenotypic switching of vascular smooth muscle cells
Source: Aging (Albany NY). 2021 Jun 8;13(11):14892–909. doi: 10.18632/aging.202998 (PMC8221363; doi:10.18632/aging.202998)
Supplement: Supplementary Table 1 [file aging-13-202998-s002.pdf]

SUPPLEMENTARY TABLE

Supplementary Table 1. Primers used for real-time qPCR assay.

Gene	Species	Primers (FW)	Primers (RW)
TNF-α	Human	CCCAGGGACCTCTCTCTAATC	ATGGGCTACAGGCTTGTCACT
IL-6	Human	ACTCACCTCTTCAGAACGAATTG	CCATCTTTGGAAGGTTCAGGTTG
α-SMA	Human	GTCCCAGACATCAGGGAGTAA	TCGGATACTTCAGCGTCAGGA
Vimentin	Human	GACGCCATCAACACCGAGTT	CTTTGTCGTTGGTTAGCTGGT
OPN	Human	CGCAGACCTGACATCCAGTA	GGAAAGCGAGGAGTTGAATG
β-actin	Human	CCTGGCACCCAGCACAAT	GCCGATCCACACGGAGTACT
TNF-α	Mouse	TGATCCGCGACGTGGAA	ACCGCCTGGAGTTCTGGAA
IL-6	Mouse	GAGGATACCACTCCCAACAGACC	AAGTGCATCATCGTTGTTCATACA
β-actin	Mouse	CCGTGAAAAGATGACCCAGA	TACGACCAGAGGCATACAG
